# Deciphering Vascular Smooth Muscle Cell Phenotype Switching via Integrated Single-Cell Transcriptomics and Machine Learning: *CX3CR1* and *COL1A1* as Novel Diagnostic Biomarkers for Atherosclerosis

**DOI:** 10.3390/metabo16100742

**Published:** 2026-10-01

**Authors:** Zhiqiang Wang, Zhenyu Zhao, Zhixi Hu, Jiahao Ye

**Affiliations:** College of Chinese Medicine, Hunan University of Chinese Medicine, Changsha 410208, China; 202401020228@stu.hnucm.edu.cn (Z.W.); shizhaozhenyuya@gmail.com (Z.Z.)

**Keywords:** atherosclerosis, vascular smooth muscle cell, single-cell transcriptomics, myeloid infiltration, extracellular matrix, machine learning, *CX3CR1*, *COL1A1*

## Abstract

**Background:** Atherosclerosis is a chronic inflammatory disease of the arterial wall in which immune-cell accumulation and vascular stromal remodeling jointly shape plaque progression and stability. Because bulk-tissue transcriptomes combine signals from multiple cell types, the cellular origin of candidate expression markers must be resolved before they can be interpreted as vascular smooth muscle cell (VSMC)-intrinsic features. **Methods:** We integrated the GSE309462 human carotid-plaque single-cell RNA-sequencing dataset with the bulk arterial-tissue datasets GSE100927 and GSE57691. Intersection-derived genes were prioritized using LASSO, random forest, and SVM-RFE. Discriminatory direction was standardized for receiver operating characteristic analysis, the five consensus genes were evaluated in the independent plaque-stage dataset GSE28829, and their cellular origins were audited across 99,027 cells from 12 donors. **Results:** Forty-nine genes were shared between the SMC-cluster-associated gene set and the bulk arterial-tissue differential-expression results. Five genes (*MPEG1, CX3CR1, SERPINA3, LMCD1, and COL1A1*) were selected by all three machine-learning procedures. In the training data, direction-standardized AUCs ranged from 0.859 to 0.922. In GSE28829, *MPEG1, CX3CR1,* and *COL1A1* showed higher expression in advanced plaques and AUCs of 0.904, 0.827, and 0.928, respectively; *LMCD1* was higher in early plaques (AUC 0.870), whereas *SERPINA3* showed limited discrimination (AUC 0.442). Single-cell localization showed that *MPEG1* and *CX3CR1* were predominantly expressed in macrophages, whereas *LMCD1* and *COL1A1* were enriched in VSMCs and other vascular stromal cells. **Conclusion:** The expression signature captures compartmentally distinct processes, particularly myeloid-cell infiltration and extracellular-matrix remodeling. These genes should therefore be interpreted as plaque-associated tissue markers and mechanistic candidates rather than validated non-invasive diagnostic biomarkers or definitive VSMC-lineage markers.

## 1. Introduction

Atherosclerosis (AS) is a chronic inflammatory disease of the arterial wall and the principal pathological substrate of many cardiovascular diseases (CVDs) [1]. Global estimates attribute approximately one-third of deaths to CVD [2]. Endothelial dysfunction, lipid retention, innate and adaptive immune activation, cell death, and extracellular-matrix (ECM) remodeling interact throughout plaque development [3,4,5]. Current imaging and circulating biomarkers estimate systemic risk or established disease but do not directly resolve the cellular programs operating within an individual plaque [6]. Tissue transcriptomics can help define these programs, although surgically obtained arterial tissue is not suitable for routine screening.

There has been a notable change in understanding the transdifferentiation of vascular smooth muscle (VSMC) into various phenotypes. Historically, they were recognized as contractile cells that maintain the normal structure of blood vessels, but recent studies show that they can switch their phenotype to various pathological states, particularly in the development of AS. During AS, VSCMs undergo phenotypic switching and lose their contractile function to adopt characteristics similar to other cell types, like macrophages and foam cells, which promote inflammation [7]. A revolutionary study revealed that a portion of the immune cells present in the plaque microenvironment were actually VSMCs that had undergone such transformations [8]. The bulk RNA-seq studies that have previously dominated research largely fail to capture the highly diverse characteristics of these changes in VSMC phenotypes, as they do not account for individual cells [9]. The inflammatory signaling pathway is associated with the pathology of AS, and the inflammatory cytokines include TLR and NLRP3, suggesting that targeting inflammatory pathways may be the key to an early therapeutic strategy [10,11,12]. Additionally, the adaptation response based on specific antigens can validate disease progression, which can also be applied to immune response and the treatment of AS [13]. Single-cell RNA sequencing (scRNA-seq) is extensively used in immunology to create high-resolution atlases of immune cell landscapes, identifying heterogeneous subpopulations of macrophages and T-cells [14]. Nevertheless, due to a predominant focus on an immuno-centric perspective, this strategy has inherent limitations and ignores the significant plasticity and unique immunomodulatory potential of stromal cells such as VSMCs in the local inflammatory environment [15,16,17].

Machine learning is regarded as a powerful tool in the risk assessment and early warning of cardiovascular illnesses because of its unparalleled capacity to process large-scale, high-dimensional, and complex multiomics datasets [18]. However, according to the study conducted by Meder et al. [19], they discovered that existing diagnostic models primarily focus on the observable features in clinical settings, neglecting the significant influence that the local immune microenvironment exerts on the progression of the disease [20]. Recognizing these limitations, we incorporated immune pathway enrichment and consensus clustering for the local immune microenvironment with the marker candidates, enabling us to identify potential subgroups within the AS cohort. Bridging the translation gaps between highly detailed single-cell discoveries and broader clinical diagnostics remains a major challenge.

**Figure 1:**
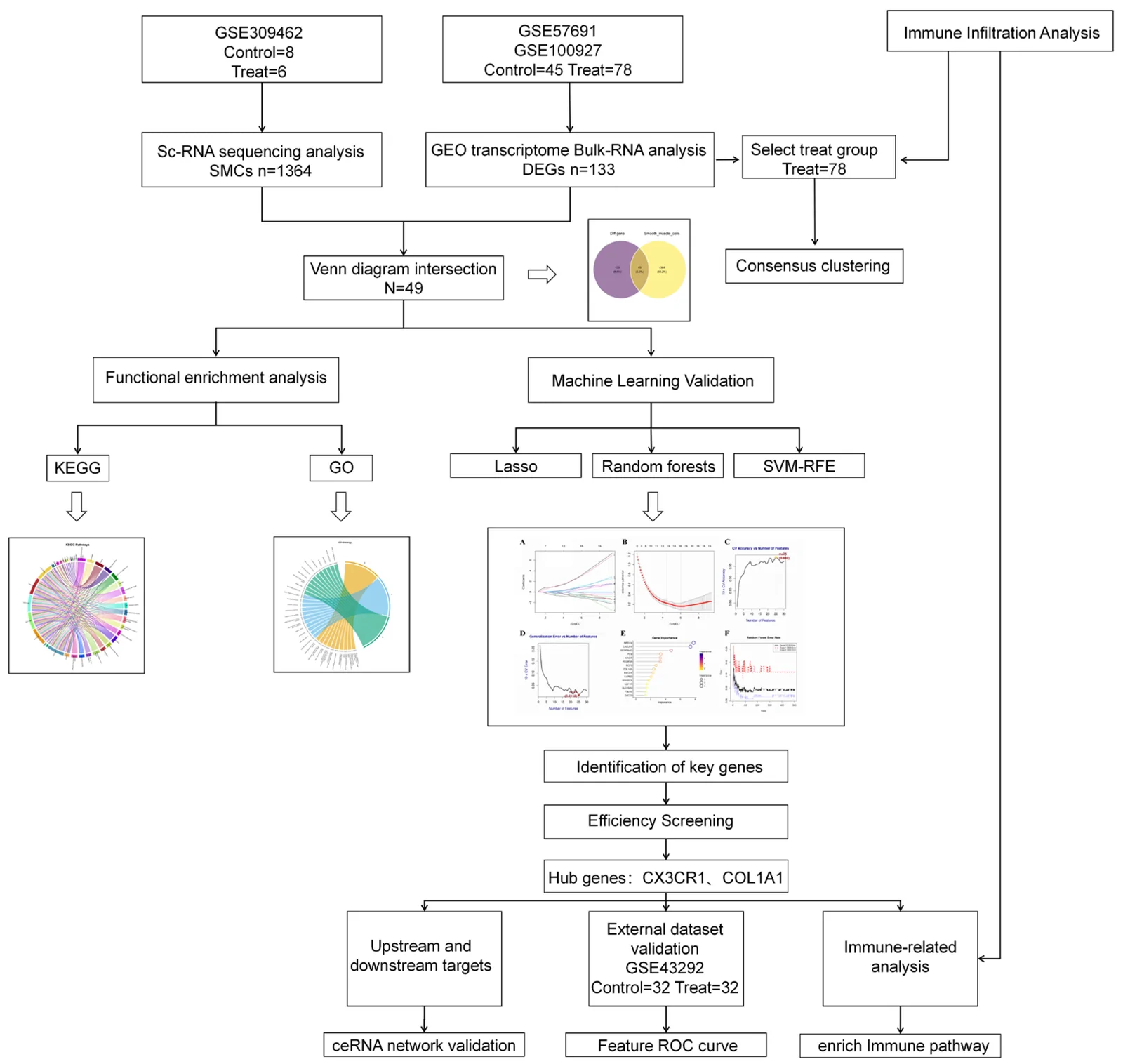
Overall Workflow of the Study.

Accordingly, we combined single-cell and bulk arterial-tissue transcriptomics with three feature-selection methods to identify plaque-associated expression features, evaluate their performance across datasets, and determine their major cellular sources. We specifically tested whether immune-associated candidates represented VSMC phenotypic remodeling or myeloid-cell infiltration and framed the resulting signals as tissue-level mechanistic markers rather than non-invasive clinical biomarkers. The overall study design and analytical workflow are summarized in Figure 1.

## 2. Methods

### 2.1. Single-Cell Transcriptomic Analysis

#### 2.1.1. Acquisition of Single-Cell Transcriptomic Data

Four public datasets were analyzed. GSE309462 contains human carotid atherosclerotic plaques from six symptomatic and six asymptomatic donors; two non-human samples in the series were excluded. GSE100927 contains 35 non-atherosclerotic control arteries and 69 atherosclerotic arteries from carotid, femoral, and infrapopliteal territories. GSE57691 contains 10 control abdominal aortas and 58 diseased aortic tissues (49 abdominal aortic aneurysms and 9 aortic occlusive-disease specimens). The integrated bulk discovery matrix therefore comprised 45 controls and 127 diseased arterial tissues. GSE28829, comprising 13 early and 16 advanced human carotid plaques, was used for independent plaque-stage validation. Because disease phenotype and vascular bed differ across datasets, these sources were recorded explicitly and addressed in sensitivity analyses and limitations.

#### 2.1.2. Processing and Analysis of scRNA-Seq Data

The deposited 10× count matrices from the 12 human GSE309462 donors were processed in R 4.4.3 using Seurat 5.4.0. Cells were retained when they contained 200–4000 detected genes, no more than 50,000 UMIs, less than 5% erythrocyte-gene counts, and less than 20% mitochondrial-gene counts. Doublets were identified separately for each donor using DoubletFinder 2.0.6. After normalization, 3000 highly variable genes were selected; the first 30 principal components were integrated with Harmony 1.2.4, using donor as the batch variable. Clustering used a shared nearest-neighbor graph at resolution 0.6, and UMAP was used for visualization. Cell identities were assigned from canonical markers and lineage-level SingleR evidence, followed by refinement of VSMC and myeloid compartments. The final object contained 99,027 cells from 12 donors. The original discovery workflow yielded 1364 genes associated with the annotated SMC cluster; this list was subsequently audited against the refined all-donor cell atlas.

#### 2.1.3. Batch Effect Correction and Differential Expression Analysis of Bulk Transcriptomic Data

GSE100927 and GSE57691 were merged at the gene-symbol level. Platform-associated effects were reduced with the removeBatchEffect function in limma 3.62.2; the corrected matrix comprised 45 control and 127 diseased arterial tissues. Differential expression was estimated with limma using the contrast diseased tissue minus control. Genes with BH-adjusted *p* < 0.05 and |log2 fold change| > 1 were considered significant. The 133 bulk-tissue DEGs were intersected with the 1364 SMC-cluster-associated genes, yielding 49 candidates. Complete mean expression, fold-change, *p*-value, and adjusted-*p*-value data are provided in Supplementary Table S1. GO and KEGG over-representation analyses used the detectable genes in the bulk matrix as the background universe.

### 2.2. Performance Validation of the Machine-Learning-Derived Diagnostic Model

The 49 intersection-derived candidates were analyzed by three feature-selection procedures. Binomial LASSO was fitted with glmnet 4.1-10 using 10-fold cross-validation. SVM-RFE used e1071 1.7-17 and caret 7.0-1 and ranked features across repeated cross-validation iterations. A random forest with 500 trees was fitted using randomForest 4.7-1.2 and ranked genes by mean decrease in Gini impurity. Genes retained by all three procedures were defined as a consensus feature set. Internal ROC, calibration, and decision-curve analyses were performed on the same discovery matrix and are therefore reported as apparent, exploratory performance rather than unbiased external validation. For individual-gene ROC analysis, AS/diseased tissue was the positive class; predictors that were lower in diseased tissue were sign-inverted so that all AUCs represented discrimination in a consistent direction. AUCs are reported with 95% confidence intervals.

### 2.3. Profiling of Immune Infiltration and Associated Pathways

Single-sample gene set enrichment analysis (ssGSEA; GSVA 2.0.7) was used to calculate relative activity scores for 15 prespecified atherosclerosis-related gene sets. Because these sets include inflammatory, apoptotic, angiogenic, and muscle-function programs, they are referred to as pathway-activity scores rather than immune-cell infiltration fractions. Scores were not interpreted as compositional proportions. Group differences were tested with two-sided Wilcoxon rank-sum tests followed by BH correction. Spearman correlations were calculated between pathway scores and *CX3CR1* or *COL1A1* expression across the 172 bulk tissues.

### 2.4. Consensus Clustering Analysis

ConsensusClusterPlus 1.70.0 was applied to the 127 diseased bulk arterial tissues using the 49 intersection-derived genes. The number of clusters was selected from consensus-matrix stability and cumulative-distribution diagnostics. PCA was used to visualize separation, and subtype-associated gene-expression and pathway-activity differences were summarized as effect directions. These subtypes are exploratory bulk-tissue states and do not represent purified VSMC subtypes.

### 2.5. Single-Cell Localization Analysis of Candidate Gene Expression

Cellular localization of *MPEG1*, *CX3CR1*, *SERPINA3*, *LMCD1*, and *COL1A1* was evaluated in the refined 99,027-cell GSE309462 object. Mean normalized expression and the fraction of positive cells were calculated across 12 major cell types together with canonical VSMC markers (ACTA2, MYH11, and TAGLN) and myeloid markers (LST1, TYROBP, and C1QA). Expression was then compared between VSMCs and macrophages using each of the 12 donors as an independent biological replicate. Paired Wilcoxon signed-rank tests were followed by BH correction.

## 3. Results

### 3.1. Single-Cell Transcriptomic Landscape of Atherosclerotic Lesions

**Figure 2:**
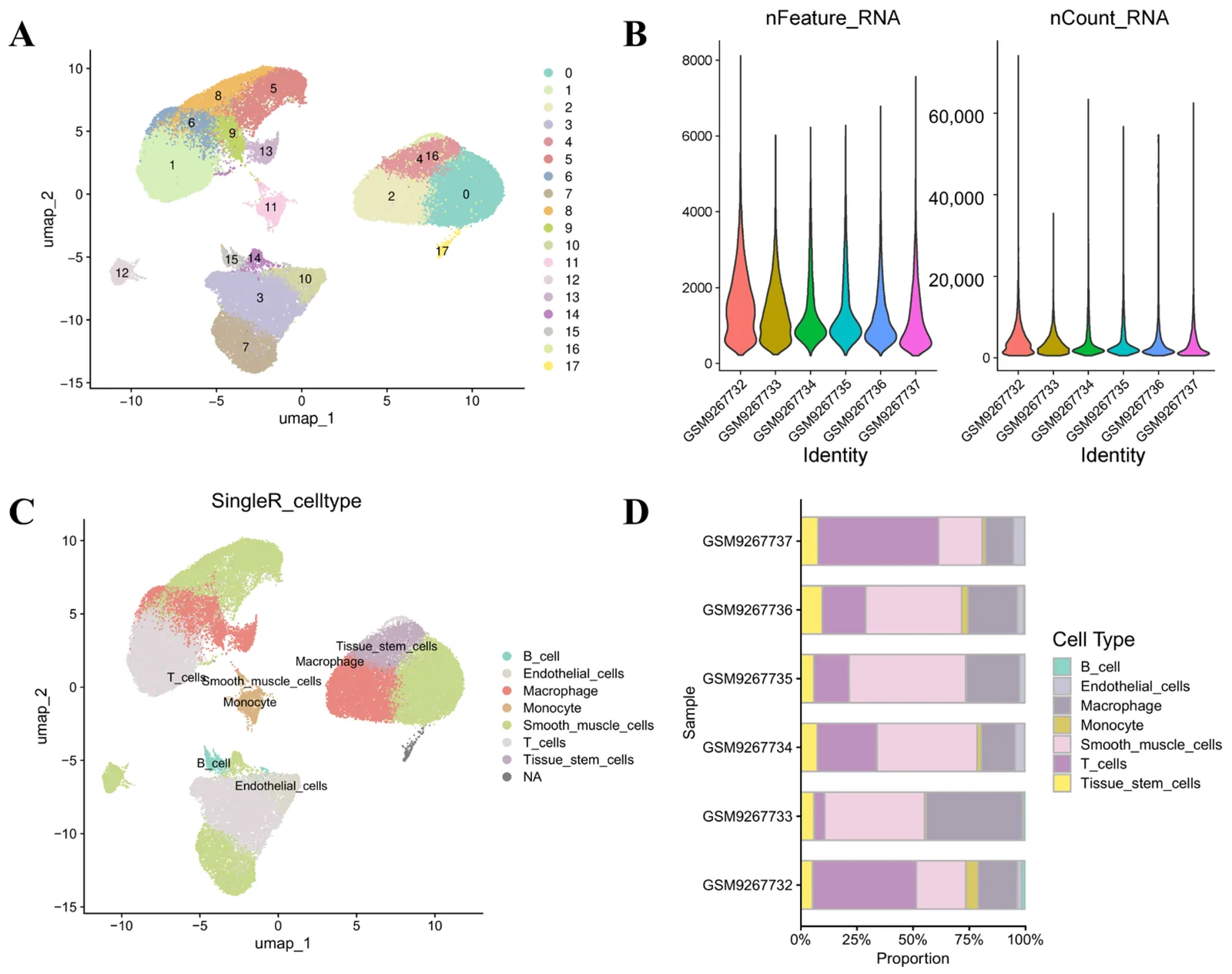
Cellular annotation and subpopulation identification within atherosclerotic samples. (**A**) UMAP scatter plot illustrating the spatial distribution of 18 distinct cellular subpopulations. (**B**) Violin plots depicting the distributions of identified gene counts and total transcript numbers across individual samples. (**C**) UMAP visualization displaying the classification and annotation of the identified cellular lineages. (**D**) Stacked bar charts representing the relative proportional composition of cell types across the distinct sequencing samples.

We preprocessed our atherosclerosis SC RNA-Seq data (GSE309462) by keeping only genes that had been expressed in a minimum of five cells and cells with at least 300 detectable transcripts to guarantee the quality of our data. As shown in Figure 2A, we used a resolution of 0.5 to cluster the subpopulations. Figure 2B shows the distribution of detected genes (nFeature) and UMI numbers (nCount) for each sample. All seven cell types from these clusters, namely, B cells, endothelial cells, macrophages, monocytes, smooth muscle cells (SMCS), T cells, and tissue stem cells (TSC), were identified using the automated annotation method (Figure 2C). Additional quality control with the thresholds 100 < nFeature _RNA < 5000, nCount _RNA > 100 was carried out. The proportional distribution of the cell types in different atherosclerotic samples was measured, and the smooth muscle cell (SMC) subpopulation was notably abundant in the AS population, leading to the detection of 1364 SMC genes.

### 3.2. Analysis of SMC-Associated Differentially Expressed Genes

**Figure 3:**
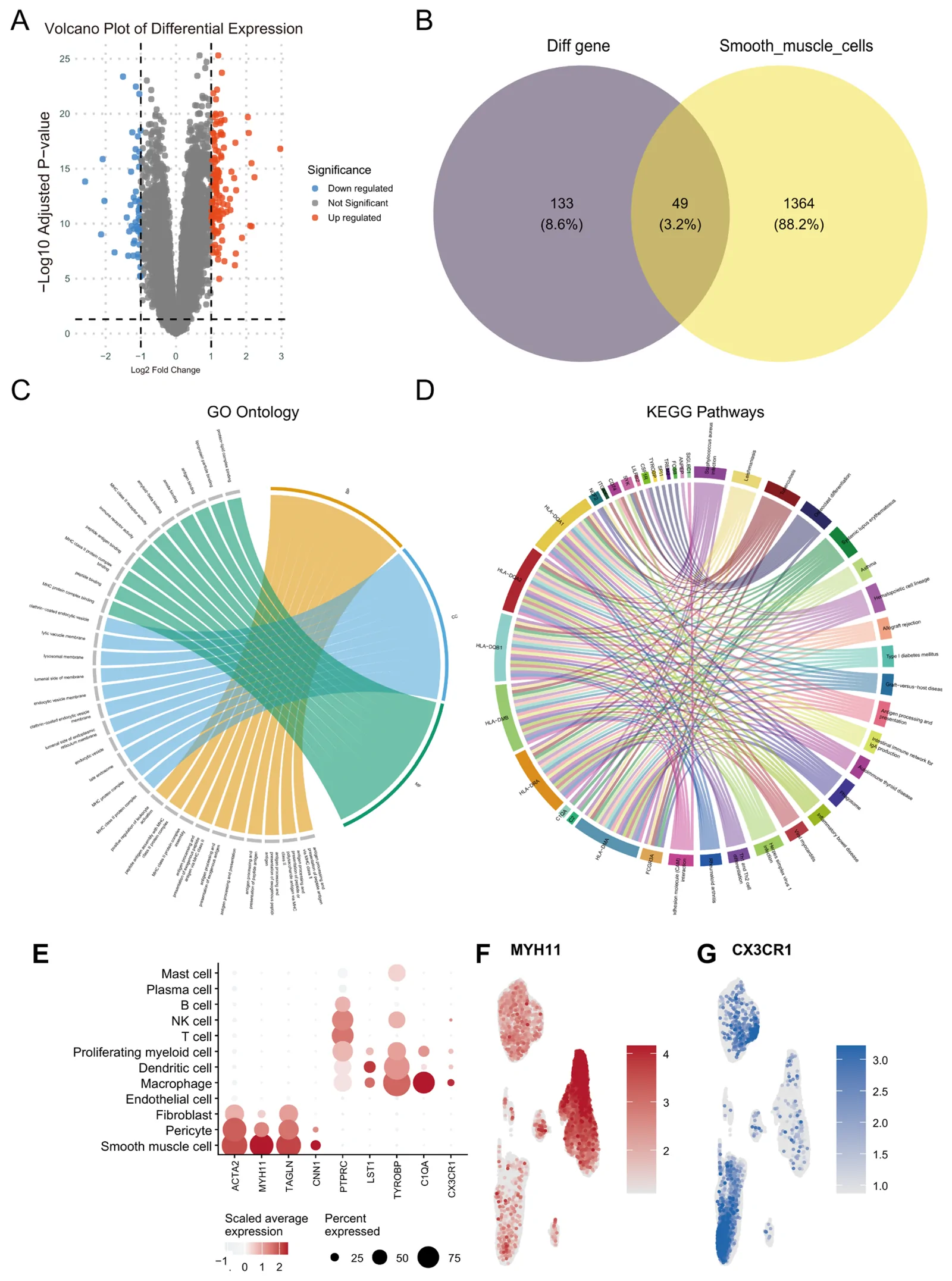
Analysis of differentially expressed genes and pathways in atherosclerosis. (**A**) volcano plot showing DEGs among atherosclerotic patients and normal controls. Up-regulated genes are depicted in red, down-regulated genes are depicted in yellow, and non-significant genes are depicted in gray. (**B**) A Venn diagram showing the overlap between the SMC-related gene set and atherosclerotic DEGs; there were 49 overlapping genes. (**C**) GO function enrichment analysis for overlapping DEGs. (**D**) KEGG pathway enrichment analysis for overlapping DEGs. (**E**) Expression patterns of VSMC and myeloid cell marker genes across the major cell types. (**F**) UMAP visualization of MYH11 expression. (**G**) UMAP visualization of *CX3CR1* expression.

Using differentially expressed gene analysis on the integrated bulk transcriptomic dataset (GSE57691 & GSE100927), we investigated the pathological relationship between SMC-associated genes and AS. In this analysis, we discovered that there were 133 differentially expressed genes when comparing atherosclerotic samples to non-atherosclerotic tissues (Figure 3A). They were then intersected with the 1364 SMC-associated genes from the previously reported single-cell RNA sequencing (Figure 3B). Then, they were narrowed down to 49 SMC-specific DEGs. The list of the core genes included *COL1A1*, *CX3CR1*, *MNDA*, *FCGR3A*, *SIGLEC1*, *PLN*, *LILRB2*, *CSF1R*, *ADAP2*, *ANPEP*, *NCF2*, *STAB1*, *HLA-DMA*, *NEXN*, *C2*, *TMEM47*, *IGFBP6*, *ITGAX*, *TYROBP*, *FOLR2*, *SLC15A3*, *MS4A4A*, *FCGR1B*, *CD74*, *HLA-DMB*, *HLA-DQB1*, *CRYAB*, *PARM1*, *DACT3*, *LY86*, *SYK*, *NFIL3*, *FBLN1*, *HLA-DQA2*, *TREM2*, *SLCO2B1*, *SPARCL1*, *RGS5*, *HLA-DQA1*, *CNN1*, *APOE*, and *FOSB*.

To improve understanding of the biological functions driving these differences, we performed GO and KEGG analyses. GO revealed that most of the DEGs were involved in processes such as antigen presentation pathways and MHC class II receptor activity [21]. These functions are located in or around certain MHC proteins and various endosomal–lysosomal compartments (Figure 3C). Moreover, KEGG analysis revealed that many of the DEGs were involved in pathways related to the antigen processing and presenting system, as well as the complement [22] and clotting systems [23]. All of these systems are related to conditions associated with infection (Figure 3D). Canonical VSMC and myeloid markers were examined to validate the cell annotation (Figure 3E). MYH11 was mainly expressed in smooth muscle cells (Figure 3F). *CX3CR1* was predominantly expressed in macrophages (Figure 3G).

### 3.3. Development and Evaluation of the Diagnostic Model

**Figure 4:**
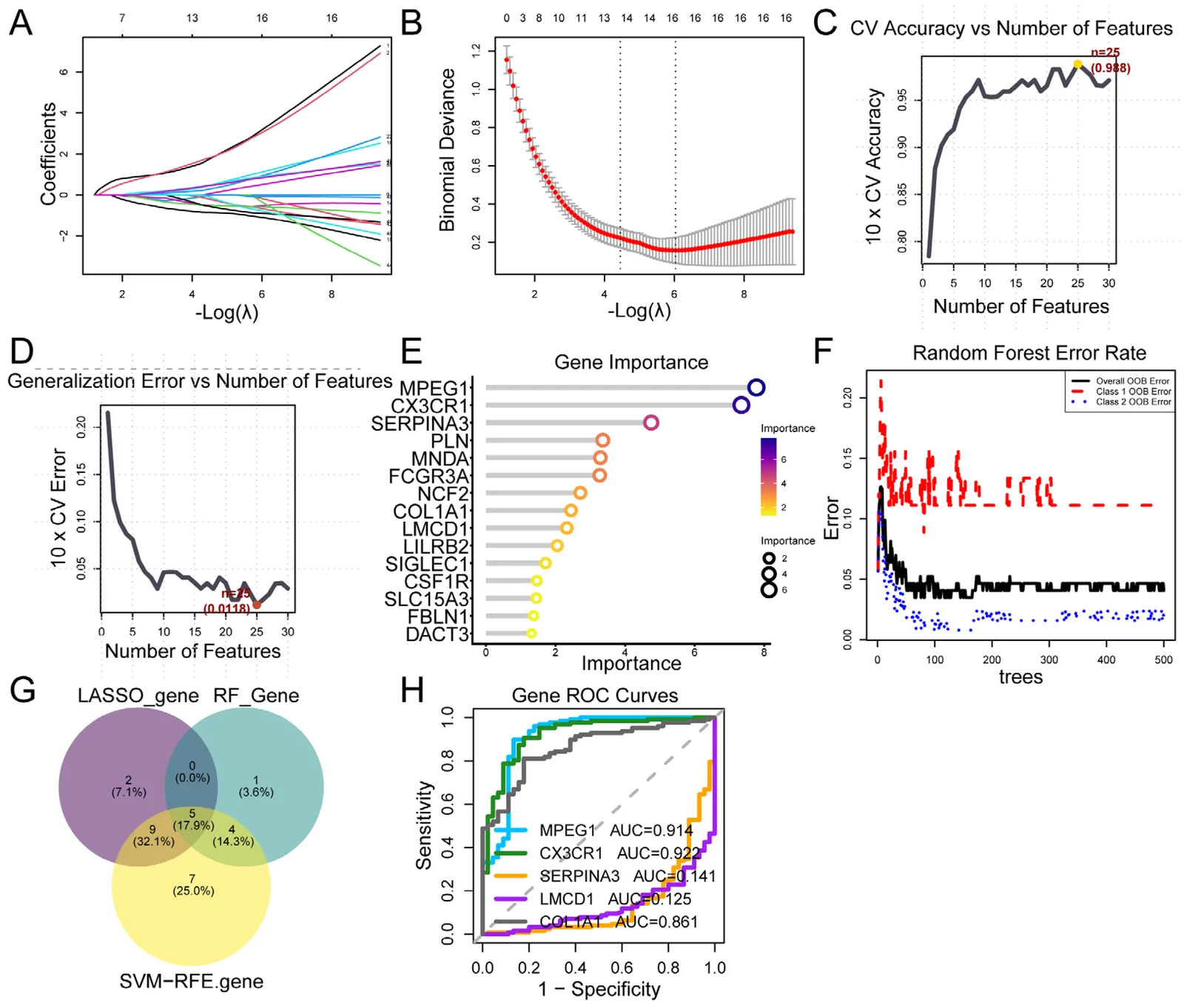
Machine learning analyses and intersections of key genes, (**A**) Coefficient trajectories of feature gene changes according to log (lambda) in LASSO models. (**B**) Cross-validation curve of binomial deviances of LASSO models. (**C**) Cross-validation accuracies plotted against the number of features in the SVM-RFE models. (**D**) The cross-validation error curve for the SVM-RFE model. (**E**) Dot chart of Random Forest analysis displaying the importance scores of the candidate genes from the Random Forest model. (**F**) Error rate convergence curve of the Random Forest model with respect to the number of decision trees. (**G**) Venn diagram showing the intersection between the five consensus hub genes found by all three algorithms. (**H**) ROC curves and AUC values for the five consensus key genes; ROC curves; the dashed diagonal line indicates the reference performance of a random classifier (AUC = 0.5).

An atherosclerosis diagnostic model using enhanced machine learning techniques was developed. LASSO Regression Analysis: As illustrated in Figure 4A,B, we initially performed a LASSO regression analysis to select features/dimensions for higher-dimensional data. SVM-RFE analysis revealed that the combination of 25 candidate genes into a diagnostic model resulted in minimal error and achieved the highest accuracy of 0.988 (Figure 4C,D). Furthermore, RF analysis identified 10 key biomarkers with significant scores greater than 3, including *MPEG1*, *CX3CR1*, *SERPINA3*, *PLN*, *MNDA*, *FCGR3A*, *NCF2*, *COL1A1*, *LMCD1*, and *LILRB2* (Figure 4E,F). Using Venn diagrams, we selected five common genes (*MPEG1*, *CX3CR1*, *SERPINA3*, *LMCD1*, and *COL1A1*) to create a consensus diagnostic framework (Figure 4G). The ROC curve analysis indicated that *MPEG1*, *CX3CR1*, and *COL1A1* exhibited high diagnostic performance, with AUC scores of 0.914, 0.922, and 0.861, respectively. In contrast, *SERPINA3* and *LMCD1* had significantly lower AUC scores of 0.141 and 0.125, respectively, indicating a notable decrease in expression levels with the onset of the disease. As a result, *MPEG1*, *CX3CR1*, and *COL1A1* were identified as the core diagnostic markers (Figure 4H).

**Figure 5:**
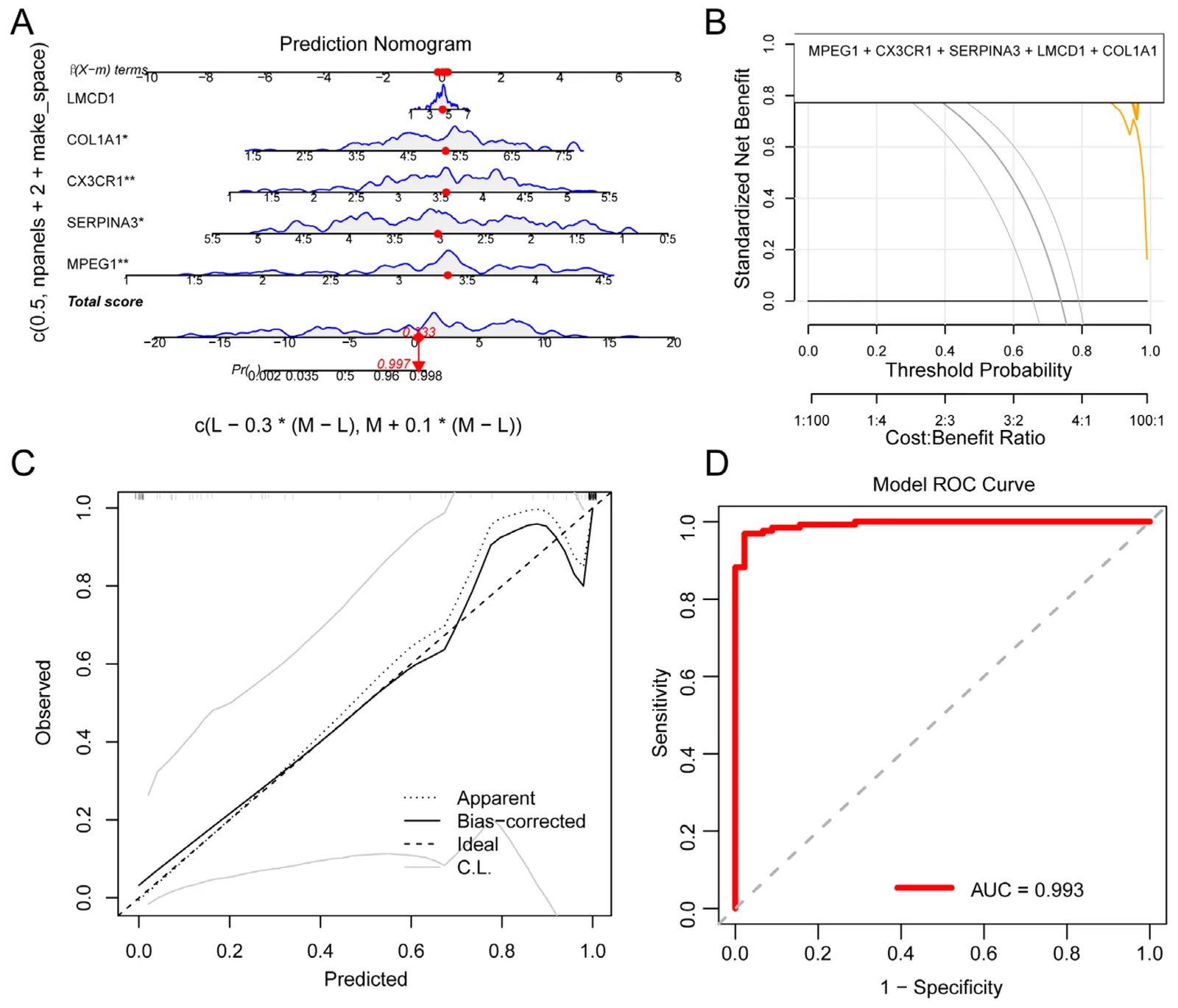
Diagnostic model validation and performance assessment. (**A**) Nomogram predicting the risk of atherosclerosis from key gene expressions. (**B**) Decision curve analysis (DCA) of the prediction model. (**C**)The calibration plot showing very similar numbers for both the observed and anticipated outcomes regarding the risk of atherosclerosis. (**D**) The ROC curve demonstrating its capability to effectively differentiate between cases that exhibit atherosclerosis; Yellow line: five-gene model; gray line: treat-all strategy; dashed line: reference line. * indicates *p* < 0.05, and ** indicates *p* < 0.01.

We also developed a nomogram for use in clinical settings (Figure 5A). The calibration graph showed little discrepancy between real and projected atherosclerosis risks, indicating high accuracy of the models (Figure 5B). As shown in Figure 5C, DCA discovered a significant improvement in the clinical value of the net benefit of the model, indicating its potential clinical use in decision making. The ROC curve analysis verified the robust and outstanding predictive ability of the combined model (Figure 5D).

### 3.4. Evaluation of Diagnostic Efficacy and External Validation

**Figure 6:**
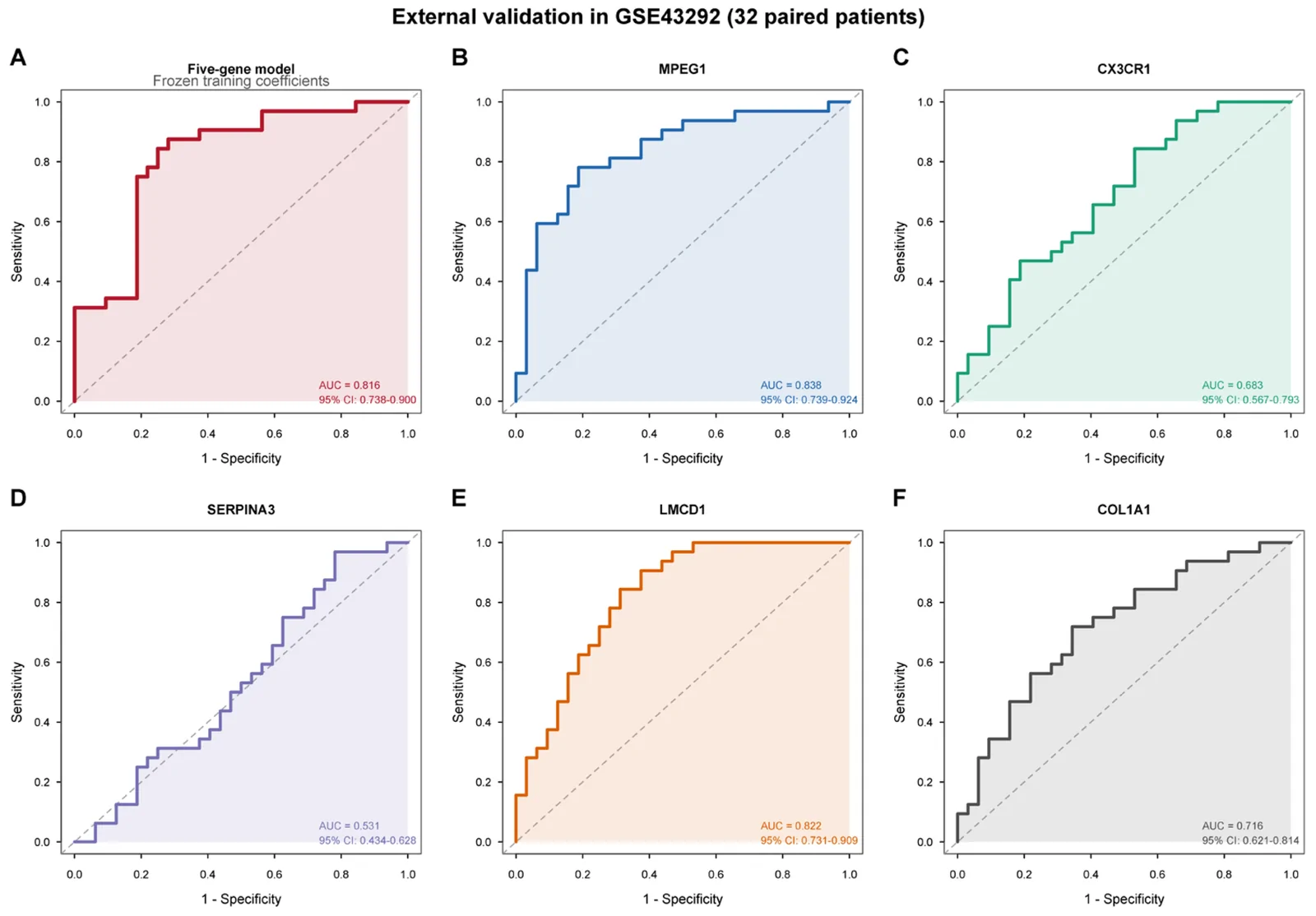
External validation of the candidate genes and the combined diagnostic model in GSE43292. (**A**) ROC curve of the five-gene model constructed using coefficients fixed in the original training cohort (AUC = 0.816). (**B**) ROC curve for MPEG1 (AUC = 0.838). (**C**) ROC curve for *CX3CR1* (AUC = 0.683). (**D**) ROC curve for *SERPINA3* (AUC = 0.531). (**E**) ROC curve for *LMCD1* (AUC = 0.822). (**F**) ROC curve for *COL1A1* (AUC = 0.716). GSE43292 comprised paired atherosclerotic plaque and macroscopically intact arterial tissue samples from 32 patients. The 95% confidence interval for each AUC is shown.

The five candidate genes and the combined diagnostic model were externally validated in the independent GSE43292 dataset, which comprised paired atherosclerotic plaque and macroscopically intact arterial tissues from 32 patients. To prevent information leakage caused by model refitting in the validation cohort, the five-gene model score was calculated using coefficients prespecified in the original training cohort. ROC analysis yielded an AUC of 0.816 (95% CI, 0.738–0.900), indicating good discriminatory performance in the independent cohort (Figure 6A).

Subsequently, we comprehensively evaluated the diagnostic performance of all five candidate genes individually, without selectively excluding genes that performed poorly in the external validation cohort. *MPEG1* showed good discriminatory performance, with an AUC of 0.838 (Figure 6B). *CX3CR1* yielded an AUC of 0.683, indicating relatively limited ability to independently distinguish plaque from control tissues (Figure 6C). *SERPINA3* had an AUC of 0.531, which was close to random classification and indicated little independent diagnostic value in this cohort (Figure 6D). *LMCD1* demonstrated good discriminatory performance, with an AUC of 0.822 (Figure 6E), whereas *COL1A1* showed moderate diagnostic performance, with an AUC of 0.716 (Figure 6F).

Paired differential expression analysis further showed that *MPEG1, CX3CR1*, and *COL1A1* were significantly upregulated in plaque tissues compared with macroscopically intact arterial tissues, whereas *LMCD1* was significantly downregulated and *SERPINA3* showed no statistically significant difference. Because GSE43292 comprises bulk transcriptomic profiles of heterogeneous vascular tissues, these expression differences may reflect the combined effects of immune-cell infiltration, extracellular matrix remodeling, and alterations in vascular wall cellular composition.

### 3.5. Analysis of Immune Infiltration and SMC Subtypes

**Figure 7:**
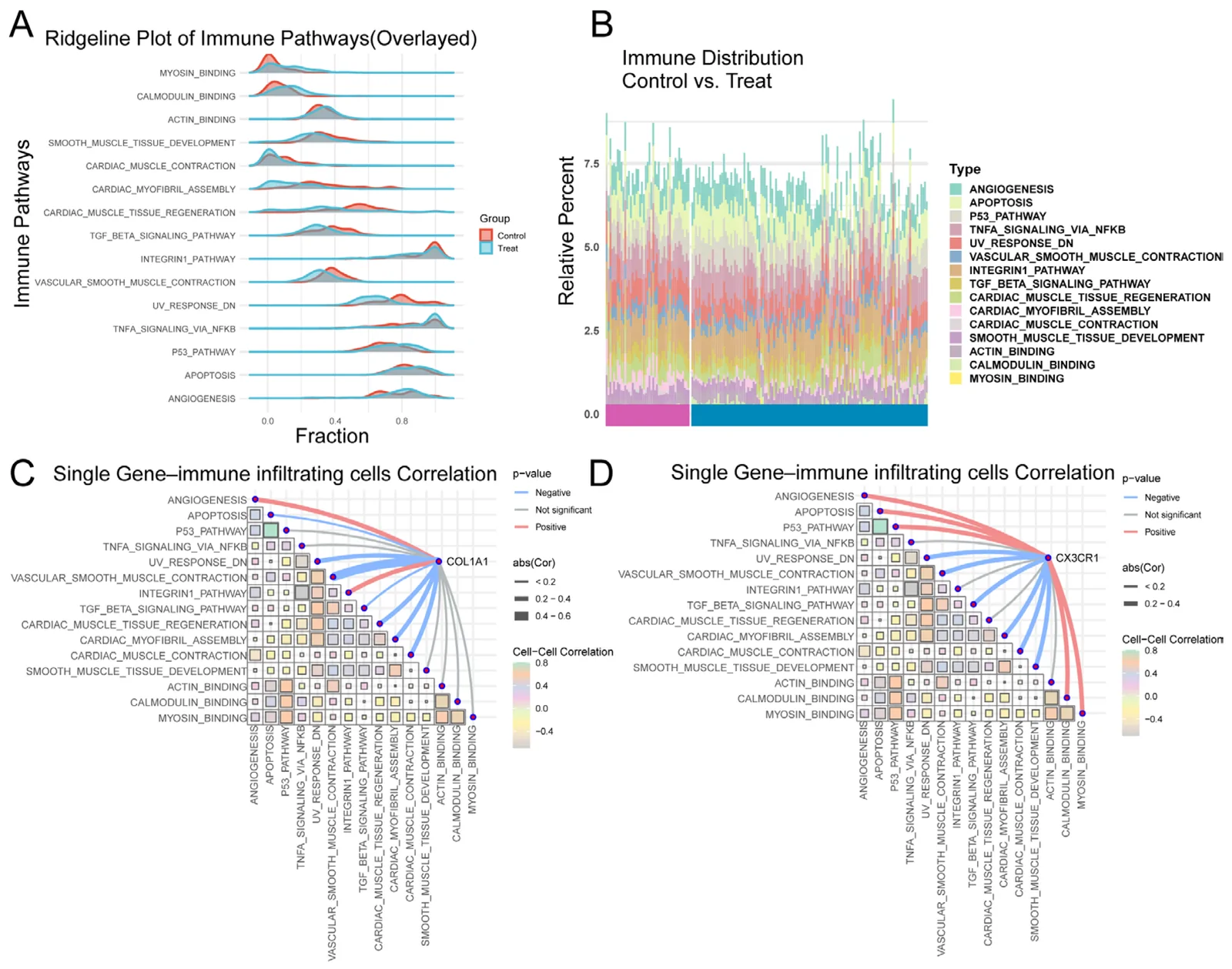
Immune pathways in atherosclerotic infiltration. (**A**) Ridgeline plot showing infiltration score distribution over immune-related pathways for the control group and AS group. (**B**) A stacked bar graph representing the proportion of different pathways for the control group vs. the AS group. (**C**) Network plot indicating the correlation between *COL1A1* expression level and the immune pathway enrichment scores (**D**) Correlation network displaying the relationship between *CX3CR1* gene expression levels and immune pathway enrichment scores. Heatmap cells are fixed-size; color indicates Spearman’s ρ, and cell size does not encode an additional variable.

For a single-sample analysis, ss gsea was performed. Furthermore, differences in immunological pathways across atherosclerotic patients were analyzed: Figure 7A,B presents a comprehensive stacked bar plot of the 15 distinct immune pathways distributed across individuals in each group, comparing the control group versus the AS group. This visualization aims to provide an understanding of the significant heterogeneity present in the local immunology across various disease states. We constructed a multidimensional correlation network (Figure 7C,D) to highlight the interaction between these signals on site and their regulatory effectors with regard to core biochemical indicators. The co-expression matrix heatmap located in the lower left section of the network illustrates significant connections among the 15 major pathways. Additionally, in relation to local immune cell infiltration, core genes exhibited a positive association with tissue remodeling processes, which included TGF-β signaling, SMC development, and angiogenesis (Figure 7C). Furthermore, we observed that *COL1A1* demonstrated strong positive correlations with other inflammatory processes, including TNF-alpha-mediated inflammatory responses, apoptosis, and UV responses as well (Figure 7D).

**Figure 8:**
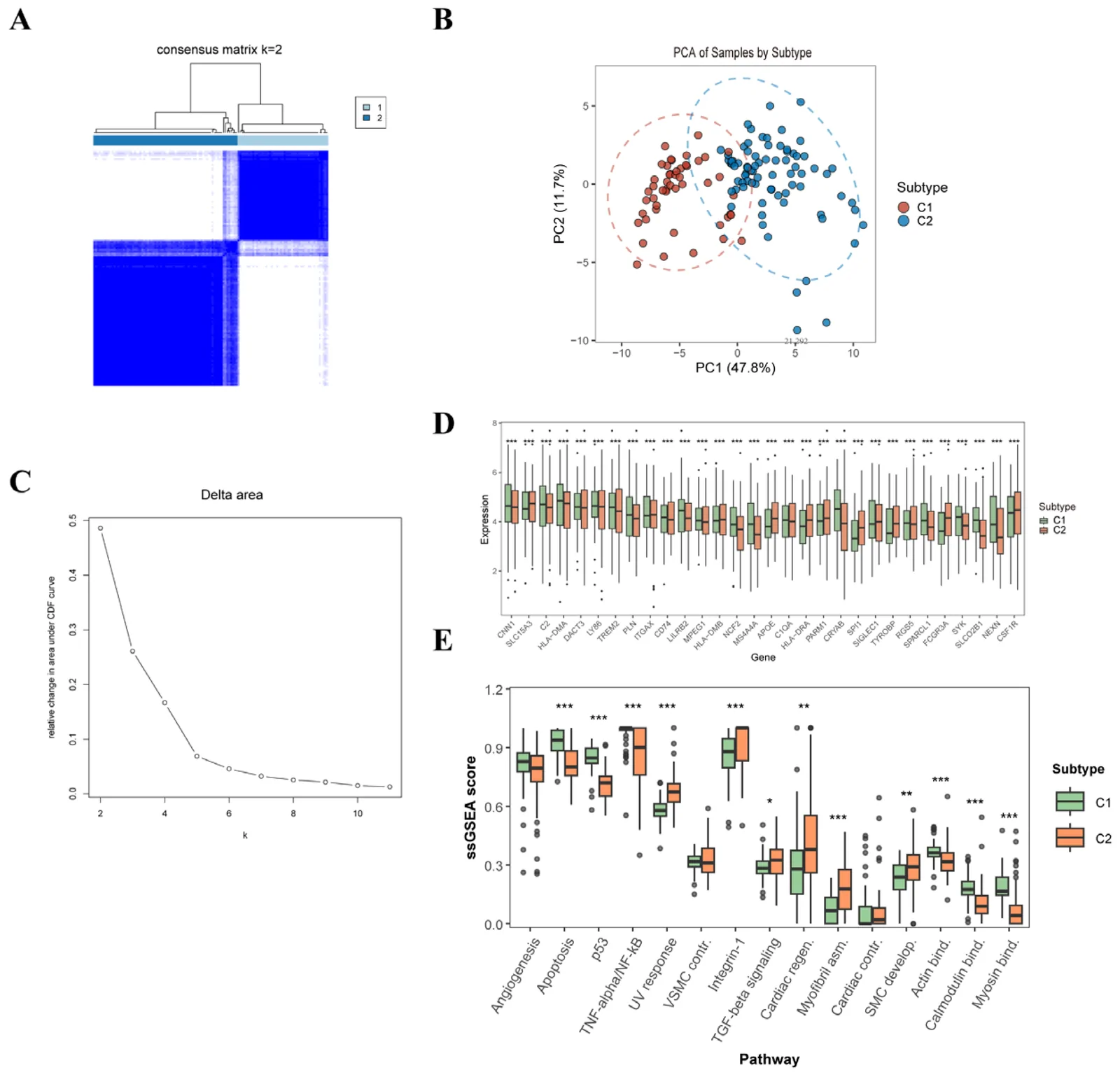
Subtypes and differences of immune infiltration. (**A**) Consensus Clustering Matrix Heatmap containing patient atherosclerotic samples (*n* = 78) to identify two different molecular subtypes. (**B**) PCA scatter plot for spatial separation of subtypes C1 and C2. (**C**) CDF Delta Area curve. (**D**) A boxplot illustrating the level of difference between these 20 SMC-associated core genes in the identified two subtypes. (**E**) Box plot representing disparity in infiltration score between various immune signal pathways and the microenvironment of Subtype C1 and Subtype C2; Statistical significance is indicated as follows: * *p* < 0.05, ** *p* < 0.01, and *** *p* < 0.001.

We performed an unsupervised consensus clustering of integrated bulk transcriptomic data of atherosclerotic patients (GSE57691, GSE100927, *n* = 78) using SMC-associated DEGs. This analysis resulted in the categorization of the data into two molecular subclasses, referred to as C1 and C2, as illustrated in Figure 8A,B. A clear clustering distinction of the two categories was observed (Figure 8C), and notable differences were observed (Figure 8D). Subtype C1 exhibited significant activation and enrichment concerning inflammation, particularly in the TNF-α-NF-κB pathway, as well as elevated activity in the apoptosis pathway (Figure 8E).

### 3.6. Cell-Type-Specific Expression Localization of the Candidate Genes

**Figure 9:**
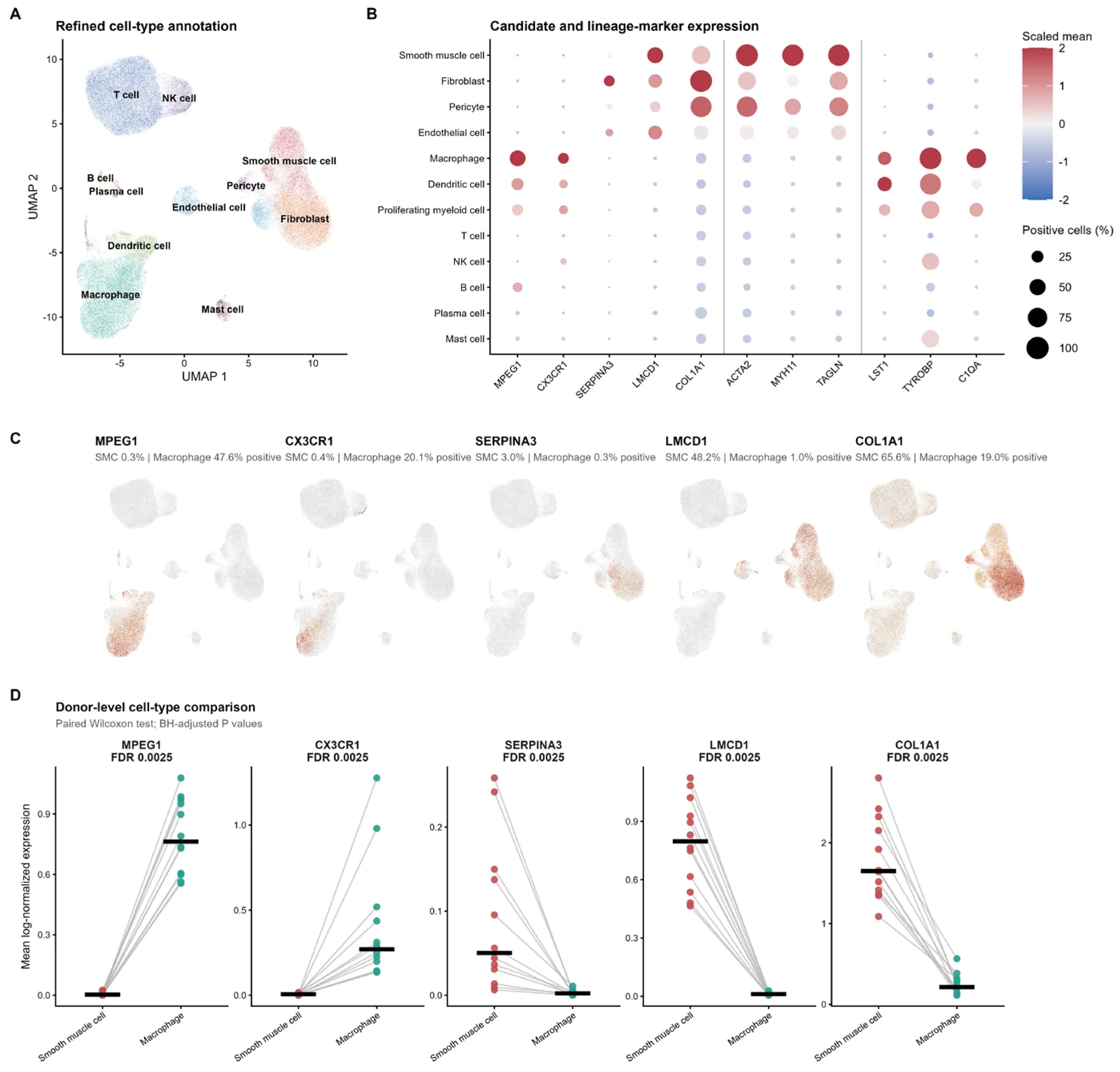
Single-cell analysis of the cell-type-specific expression patterns of the five candidate genes. (**A**) UMAP visualization of the major cell types. (**B**) Expression patterns of the candidate genes and canonical VSMC and myeloid marker genes across different cell types. (**C**) UMAP visualization of the expression distributions of the five candidate genes. (**D**) Donor-level paired comparisons of the five candidate genes between VSMCs and macrophages.

Using the GSE309462 single-cell transcriptomic dataset, we characterized the cell-type-specific expression patterns of the five candidate genes. Following marker-based validation of the cell annotations, 12 major cell types were identified (Figure 9A). Expression profiling of the candidate genes alongside canonical VSMC and myeloid markers revealed marked cell-type specificity (Figure 9B). UMAP visualization showed that *MPEG1* and *CX3CR1* were predominantly expressed in macrophages, whereas *LMCD1* and *COL1A1* were enriched in VSMCs and other vascular wall stromal cells (Figure 9C). Paired analyses using the 12 donors as independent biological replicates further demonstrated significant differences in the expression of all five candidate genes between VSMCs and macrophages (Figure 9D). Collectively, these findings indicate that *MPEG1* and *CX3CR1* primarily reflect myeloid-cell signals, whereas *LMCD1* and *COL1A1* more closely represent the transcriptional features of vascular wall stromal cells.

## 4. Discussion

This research employed single-cell transcriptomes, bulk RNA sequencing, and machine learning to identify and evaluate *CX3CR1* and *COL1A1* as potential atherosclerosis markers. According to our research, VSMCs underwent significant remodeling in relation to immunophenotypes and extracellular matrix during the progression of the disease. This finding may provide a novel perspective in understanding VSMC diversity and identifying factors that may pose risks for plaque formation. In contrast to the traditional view that VSMCs only contribute to the structural integrity of the cells, recent findings have demonstrated their remarkable plasticity and potential for dedifferentiation in AS [24,25]. Cell-type-specific expression analysis indicated that immune-associated signals, particularly *CX3CR1*, were predominantly derived from macrophages rather than VSMCs. Thus, our findings support the coexistence of immune-cell infiltration and extracellular matrix remodeling within atherosclerotic plaques.

The cell-of-origin audit directly addresses the risk of misattributing immune transcripts to VSMCs. Fewer than 0.5% of VSMCs expressed *MPEG1* or *CX3CR1*, while macrophage expression was substantially higher. Therefore, elevated bulk *CX3CR1* is more plausibly explained by macrophage abundance or activation than by VSMC conversion into a macrophage-like state [26]. Conversely, *COL1A1* expression across VSMCs, fibroblasts, and pericytes supports its interpretation as a vascular-wall matrix-remodeling signal. Co-occurrence of these genes in bulk tissue does not imply co-expression within the same cells.

The primary involvement of VSMCs in plaque vulnerability is highlighted by matrix reconfiguration in relation to *COL1A1* aberrant expression. Our immune infiltration and consensus clustering analyses demonstrated that *COL1A1* expression was positively correlated with numerous immune pathways and the over-activation of TNF-α/NF-κB and apoptotic signaling in specific VSMC subtypes, in contrast to the traditional understanding of *COL1A1* as a simple protective element of the fibrous cap. From these results, we propose a novel pathological model stating that under prolonged inflammatory stimulation, certain VSMCs attempt to compensate for the damage by synthesizing ECM components such as *COL1A1* to stabilize the plaque [27]; however, persistent inflammation ultimately leads to a failure of homeostasis and cell death [28]. Therefore, the co-occurrence of *CX3CR1* and *COL1A1* signals in bulk tissues more likely reflects immune-cell infiltration and extracellular matrix remodeling, respectively, rather than a shared activation program within the same VSMC subpopulation [29].

The external GSE28829 results support association with carotid plaque stage rather than early diagnosis of atherosclerosis. This distinction is clinically important because every discovery and validation sample was excised arterial tissue obtained during surgery or autopsy. Such material is not suitable for routine screening. Translation would require prospective evaluation in accessible biospecimens, such as blood cells, plasma proteins or extracellular vesicles, and comparison with established clinical and imaging measures.

Several limitations constrain interpretation. First, the bulk discovery datasets combine carotid, femoral, infrapopliteal, and abdominal aortic tissues; vascular smooth muscle and other vascular cells exhibit developmental and region-specific heterogeneity. Dataset, vascular bed, and disease phenotype are partly confounded, particularly because GSE57691 contains abdominal aortic aneurysm and aortic occlusive disease. Dataset-specific sensitivity results are therefore provided in Supplementary Table S5, but they cannot fully separate anatomical from cohort effects. Second, internal model metrics are optimistic because feature selection and assessment used the same matrix. Third, GSE28829 compares early with advanced plaque and does not include healthy participants. Finally, functional or prospective clinical validation was not performed.

## 5. Conclusions

Integrated transcriptomic analysis identified a tissue expression signature spanning myeloid infiltration and vascular stromal remodeling in human atherosclerotic plaques. *CX3CR1* predominantly represents a macrophage-associated signal, whereas *COL1A1* reflects ECM remodeling across vascular stromal cells, including VSMCs. These findings refine the biological interpretation of the candidate genes and generate testable mechanistic hypotheses, but they do not establish a non-invasive diagnostic assay, a validated clinical risk model, or direct therapeutic targets.

## Data Availability

The data presented in this study are openly available in Gene Expression Omnibus (GEO) at https://www.ncbi.nlm.nih.gov/geo/ reference number [GSE309462; GSE57691; GSE100927; GSE43292].

## Supplementary Materials

The following supporting information can be downloaded at: https://www.mdpi.com/article/10.3390/metabo16100742/s1; Table S1: Complete statistics for the 49 intersection-derived genes; Table S2: Functions and cellular interpretation of the 10 random-forest-ranked genes; Table S3: Abbreviations; Table S4: Software versions; Table S5: Dataset-specific sensitivity analysis of the five consensus genes.
