# Supplementary material for: Deciphering Vascular Smooth Muscle Cell Phenotype Switching via Integrated Single-Cell Transcriptomics and Machine Learning: CX3CR1 and COL1A1 as Novel Diagnostic Biomarkers for Atherosclerosis
Source: Metabolites. 2026 Oct 1;16(10):742. doi: 10.3390/metabo16100742 (PMC13706743; doi:10.3390/metabo16100742)
Supplement: Supplementary file 1 [file metabolites-16-00742-s001.zip › Supplementary_Tables.pdf]

Supplementary Materials

Integrated Single-Cell and Bulk Transcriptomics Reveals Myeloid Infiltration and Stromal Remodeling Signatures in Human Atherosclerosis

Table S1. Complete statistics for the 49 intersection-derived genes.

Gene	Control mean	Diseased mean	log2FC	P value	BH-adjusted P	Direction
MPEG1	2.5303	3.5933	1.0629	9.556e-26	1.354e-22	Higher in diseased tissue
CX3CR1	2.6519	3.8239	1.1720	1.271e-22	5.271e-20	Higher in diseased tissue
MNDA	2.6482	3.7099	1.0616	2.600e-21	5.819e-19	Higher in diseased tissue
FCGR3A	3.4104	4.6991	1.2887	3.350e-21	7.249e-19	Higher in diseased tissue
SIGLEC1	2.1767	3.2319	1.0552	1.844e-20	3.016e-18	Higher in diseased tissue
PLN	3.8376	2.6650	-1.1725	9.085e-19	8.779e-17	Lower in diseased tissue
LILRB2	3.0099	4.0675	1.0576	1.634e-18	1.400e-16	Higher in diseased tissue
CSF1R	3.9293	5.1180	1.1887	2.794e-17	1.654e-15	Higher in diseased tissue
ADAP2	3.1552	4.3810	1.2258	3.859e-17	2.202e-15	Higher in diseased tissue
ANPEP	2.2466	3.3384	1.0918	1.805e-16	8.721e-15	Higher in diseased tissue
NCF2	2.6749	3.8071	1.1322	2.577e-16	1.204e-14	Higher in diseased tissue
STAB1	3.1438	4.1560	1.0122	8.163e-16	3.550e-14	Higher in diseased tissue
HLA-DMA	3.2551	4.3009	1.0458	2.034e-15	8.046e-14	Higher in diseased tissue
NEXN	4.5781	3.4368	-1.1413	3.231e-15	1.192e-13	Lower in diseased tissue
SERPINA3	3.8665	2.6649	-1.2016	1.278e-14	4.123e-13	Lower in diseased tissue
C2	2.5491	3.6803	1.1312	1.456e-14	4.628e-13	Higher in diseased tissue
LMCD1	5.0473	4.0270	-1.0203	2.157e-14	6.563e-13	Lower in diseased tissue
TMEM47	3.6970	2.5345	-1.1625	3.057e-14	8.886e-13	Lower in diseased tissue
IGFBP6	4.7767	3.7297	-1.0471	3.534e-14	1.009e-12	Lower in diseased tissue
ITGAX	2.2977	3.3421	1.0444	3.796e-14	1.076e-12	Higher in diseased tissue
TYROBP	4.0964	5.3209	1.2245	4.123e-14	1.157e-12	Higher in diseased tissue
HMOX1	3.7524	5.2230	1.4706	7.750e-14	2.022e-12	Higher in diseased tissue
SPI1	2.8756	4.0106	1.1350	8.737e-14	2.251e-12	Higher in diseased tissue
C1QA	3.4388	4.5343	1.0955	2.078e-13	4.888e-12	Higher in diseased tissue
COL1A1	4.1998	5.4857	1.2859	2.663e-13	6.103e-12	Higher in diseased tissue
HLA-DRA	3.9699	5.0126	1.0426	4.630e-13	1.004e-11	Higher in diseased tissue
FOLR2	2.9591	4.0132	1.0541	6.195e-13	1.294e-11	Higher in diseased tissue
SLC15A3	3.1711	4.1819	1.0108	8.398e-13	1.719e-11	Higher in diseased tissue
MS4A4A	3.4331	4.4618	1.0287	9.713e-13	1.969e-11	Higher in diseased tissue
FCGR1B	2.9920	4.0106	1.0186	1.011e-12	2.024e-11	Higher in diseased tissue
CD74	3.0370	4.0550	1.0180	1.118e-12	2.213e-11	Higher in diseased tissue
HLA-DMB	3.7708	4.8554	1.0846	1.265e-12	2.462e-11	Higher in diseased tissue
HLA-DQB1	3.1347	4.3540	1.2193	1.707e-12	3.240e-11	Higher in diseased tissue
CRYAB	5.1179	4.0553	-1.0627	1.742e-12	3.300e-11	Lower in diseased tissue
PARM1	3.7420	2.7217	-1.0203	2.553e-12	4.648e-11	Lower in diseased tissue
DACT3	4.8877	3.7384	-1.1493	4.311e-12	7.498e-11	Lower in diseased tissue
LY86	3.1403	4.1468	1.0065	5.942e-12	1.001e-10	Higher in diseased tissue
SYK	2.5920	3.6333	1.0413	8.786e-12	1.434e-10	Higher in diseased tissue
NFIL3	4.5951	3.5927	-1.0024	1.260e-11	1.970e-10	Lower in diseased tissue
FBLN1	4.9846	3.9476	-1.0370	2.638e-11	3.887e-10	Lower in diseased tissue
HLA-DQA2	2.9053	3.9923	1.0870	3.861e-11	5.476e-10	Higher in diseased tissue
TREM2	2.4759	3.6538	1.1779	5.189e-11	7.151e-10	Higher in diseased tissue
SLCO2B1	2.8444	3.9100	1.0656	6.809e-11	9.242e-10	Higher in diseased tissue
SPARCL1	5.4923	4.4866	-1.0056	8.993e-11	1.186e-09	Lower in diseased tissue

Gene	Control mean	Diseased mean	log2FC	P value	BH-adjusted P	Direction
RGS5	5.4120	4.2350	-1.1770	3.147e-10	3.655e-09	Lower in diseased tissue
HLA-DQA1	3.0714	4.3000	1.2286	1.648e-09	1.609e-08	Higher in diseased tissue
CNN1	6.0398	4.8236	-1.2162	9.862e-09	8.157e-08	Lower in diseased tissue
APOE	4.3296	5.7086	1.3790	3.595e-08	2.621e-07	Higher in diseased tissue
FOSB	3.1193	4.3502	1.2309	2.090e-06	1.057e-05	Higher in diseased tissue

Mean expression values are from the batch-corrected integrated bulk matrix (45 control and 127 diseased arterial tissues). log2 fold change is diseased minus control. P values were estimated with limma and adjusted using the Benjamini-Hochberg procedure.

Table S2. Functions and cellular interpretation of the 10 random-forest-ranked genes.

Gene	Established function	Predominant cellular context	Interpretation in this study
MPEG1	Macrophage-expressed gene 1; a pore-forming innate-immune protein enriched in mononuclear phagocytes.	Macrophages	Myeloid-cell abundance/activation
CX3CR1	Fractalkine receptor regulating monocyte adhesion, trafficking and survival.	Macrophages and monocytes	Myeloid recruitment and plaque inflammation
SERPINA3	Secreted serine-protease inhibitor involved in acute-phase responses and extracellular proteolysis.	Hepatic and inflamed tissue-associated cells	Inflammation/protease balance
PLN	Phospholamban, a regulator of SERCA-dependent calcium reuptake and contractile-cell calcium homeostasis.	Muscle-lineage cells	Contractile calcium handling
MNDA	Interferon-responsive myeloid nuclear protein associated with myeloid differentiation.	Monocytes and macrophages	Myeloid inflammatory state
FCGR3A	Low-affinity Fc-gamma receptor CD16A mediating immune-complex recognition and effector signaling.	Monocytes, macrophages and NK cells	Innate immune effector activity
NCF2	Cytosolic NADPH-oxidase subunit required for phagocyte reactive-oxygen-species production.	Myeloid cells	Oxidative inflammatory activity
COL1A1	Major alpha chain of type I collagen and a central component of vascular extracellular matrix.	VSMCs, fibroblasts and pericytes	Matrix deposition and fibrous-cap remodeling
LMCD1	LIM-domain transcriptional co-regulator implicated in muscle differentiation and cardiovascular remodeling.	VSMCs and vascular stromal cells	Contractile/stromal state
LILRB2	Inhibitory leukocyte immunoglobulin-like receptor that restrains myeloid-cell activation.	Monocytes, macrophages and dendritic cells	Myeloid immune regulation

Table S3. Abbreviations.

Abbreviation	Definition
AS	atherosclerosis
AUC	area under the ROC curve
BH	Benjamini-Hochberg
CCA	canonical correlation analysis
CVD	cardiovascular disease
DCA	decision-curve analysis
DEG	differentially expressed gene
ECM	extracellular matrix
FDR	false discovery rate
GEO	Gene Expression Omnibus
GO	Gene Ontology
HVG	highly variable gene
KEGG	Kyoto Encyclopedia of Genes and Genomes
LASSO	least absolute shrinkage and selection operator
PCA	principal component analysis
RF	random forest
ROC	receiver operating characteristic
scRNA-seq	single-cell RNA sequencing
SNN	shared nearest-neighbor
ssGSEA	single-sample gene set enrichment analysis
SVM-RFE	support vector machine-recursive feature elimination
UMAP	uniform manifold approximation and projection
UMI	unique molecular identifier
VSMC	vascular smooth muscle cell

Table S4. Software versions.

Software/package	Version
R	4.4.3
Seurat	5.4.0
SeuratObject	5.3.0
Harmony	1.2.4
DoubletFinder	2.0.6
SingleR	2.8.0
limma	3.62.2
clusterProfiler	4.14.6
glmnet	4.1-10
e1071	1.7-17
caret	7.0-1
randomForest	4.7-1.2
pROC	1.19.0.1
GSVA	2.0.7
ConsensusClusterPlus	1.70.0
ggplot2	4.0.3
ggpubr	0.6.2
pheatmap	1.0.13
patchwork	1.3.2

Table S5. Dataset-specific sensitivity analysis of the five consensus genes.

Dataset/vascular context	Gene	Control n	Disease n	Control mean	Disease mean	Difference	P value
GSE100927 (carotid/femoral/infrapopliteal)	*MPEG1*	35	69	2.4041	3.7669	1.3627	3.746e-13
GSE100927 (carotid/femoral/infrapopliteal)	*CX3CR1*	35	69	2.5827	4.0033	1.4206	6.538e-13
GSE100927 (carotid/femoral/infrapopliteal)	*SERPINA3*	35	69	3.7818	2.5995	-1.1822	1.299e-09
GSE100927 (carotid/femoral/infrapopliteal)	*LMCD1*	35	69	5.0831	3.9756	-1.1076	2.927e-14
GSE100927 (carotid/femoral/infrapopliteal)	*COL1A1*	35	69	4.1902	5.7350	1.5448	2.481e-13
GSE57691 (abdominal aorta; AAA/AOD)	*MPEG1*	10	58	2.9721	3.3868	0.4147	8.929e-06
GSE57691 (abdominal aorta; AAA/AOD)	*CX3CR1*	10	58	2.8942	3.6105	0.7163	1.048e-05
GSE57691 (abdominal aorta; AAA/AOD)	*SERPINA3*	10	58	4.1631	2.7427	-1.4204	3.912e-05
GSE57691 (abdominal aorta; AAA/AOD)	*LMCD1*	10	58	4.9220	4.0883	-0.8337	0.0079
GSE57691 (abdominal aorta; AAA/AOD)	*COL1A1*	10	58	4.2336	5.1891	0.9556	0.0136

This sensitivity analysis was performed within each bulk discovery dataset to expose heterogeneity associated with vascular bed and disease phenotype. Values are normalized expression means; P values are two-sided Mann-Whitney tests and are descriptive because dataset and anatomical site remain confounded.

Supplementary Figure and source-data files

Machine-readable source tables generated during revision include the direction-corrected training ROC results, independent GSE28829 ROC results, pathway-activity group comparisons and gene-pathway correlations. Editable SVG and PDF versions and 600-dpi TIFF versions of the revised figures are supplied in the revision figure folder.
